# Social Frailty Is Independently Associated with Mood, Nutrition, Physical Performance, and Physical Activity: Insights from a Theory-Guided Approach

**DOI:** 10.3390/ijerph17124239

**Published:** 2020-06-14

**Authors:** Kalene Pek, Justin Chew, Jun Pei Lim, Suzanne Yew, Cai Ning Tan, Audrey Yeo, Yew Yoong Ding, Wee Shiong Lim

**Affiliations:** 1 Institute of Geriatrics and Active Ageing, Tan Tock Seng Hospital, Singapore 308433, Singapore; justin_chew@ttsh.com.sg (J.C.); jun_pei_lim@ttsh.com.sg (J.P.L.); suzanne_py_yew@ttsh.com.sg (S.Y.); cai_ning_tan@ttsh.com.sg (C.N.T.); audrey_jp_yeo@ttsh.com.sg (A.Y.); yew_yoong_ding@ttsh.com.sg (Y.Y.D.); 2 Department of Geriatric Medicine, Tan Tock Seng Hospital, Singapore 308433, Singapore

**Keywords:** social frailty, physical frailty, social gradient, nutrition, physical performance, physical activity

## Abstract

Notwithstanding the increasing body of evidence that links social determinants to health outcomes, social frailty is arguably the least explored among the various dimensions of frailty. Using available items from previous studies to derive a social frailty scale as guided by the Bunt social frailty theoretical framework, we aimed to examine the association of social frailty, independently of physical frailty, with salient outcomes of mood, nutrition, physical performance, physical activity, and life–space mobility. We studied 229 community-dwelling older adults (mean age 67.22 years; 72.6% females) who were non-frail (defined by the FRAIL criteria). Using exploratory factor analysis, the resultant 8-item Social Frailty Scale (SFS-8) yielded a three-factor structure comprising social resources, social activities and financial resource, and social need fulfilment (score range: 0–8 points). Social non-frailty (SNF), social pre-frailty (SPF), and social frailty (SF) were defined based on optimal cutoffs, with corresponding prevalence of 63.8%, 28.8%, and 7.4%, respectively. In logistic regression adjusted for significant covariates and physical frailty (Modified Fried criteria), there is an association of SPF with poor physical performance and low physical activity (odds ratio, OR range: 3.10 to 6.22), and SF with depressive symptoms, malnutrition risk, poor physical performance, and low physical activity (OR range: 3.58 to 13.97) compared to SNF. There was no significant association of SPF or SF with life–space mobility. In summary, through a theory-guided approach, our study demonstrates the independent association of social frailty with a comprehensive range of intermediary health outcomes in more robust older adults. A holistic preventative approach to frailty should include upstream interventions that target social frailty to address social gradient and inequalities.

## 1. Introduction

Frailty is characterized by a loss of physiological reserves, leading to increased vulnerability of the older adult with stressor events [1]. Frailty is widely regarded as a multidimensional construct with physical, cognitive, psychological, and social components. Among these dimensions, social frailty is arguably the least explored. Given the complex interplay between the dimensions of frailty and increasing appreciation of the contribution of social factors to health outcomes [2], it is not surprising that social frailty has been gaining recognition and traction in recent years.

**Figure 1:**
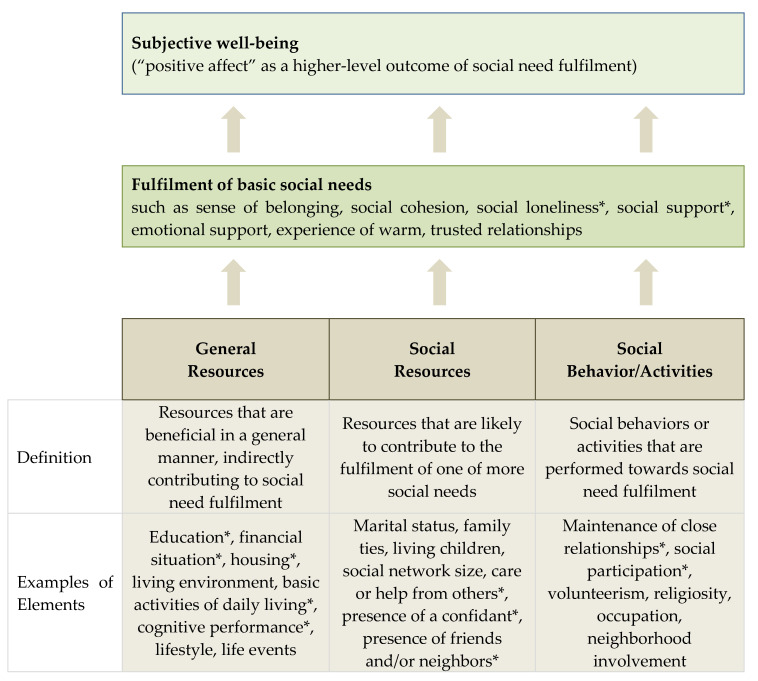
Conceptual model of social frailty proposed by Bunt et al. [5]. This figure, adapted from Bunt’s social frailty concept, shows the categories of ‘general resources’, ‘social resources’, and ‘social behavior/activities’ that lead to the ‘fulfilment of basic social needs’, which in turn lends a positive impact to subjective well-being when needs are met. Examples of elements in the categories are included, some of which are included in this study. * denotes elements included in our analysis.

However, the study of social frailty has been far from straightforward, being often intertwined with contextual, societal, and cultural considerations. The field has been hampered by the lack of theoretical frameworks to guide the conceptualization of social frailty. Using the theory of Social Production Function (SPF) [3,4], Bunt et al. recently proposed a conceptual framework whereby social frailty is defined as a continuum of being at risk of losing, or having lost, social resources, general resources, and social activities or abilities that are important for fulfilling one or more basic social needs during the life span (Figure 1) [5]. Subsequent to this, a systematic review studying the operationalization of the social component of frailty revealed only three exclusive social frailty tools out of 27 frailty instruments, and a weight of 5–43% for the social dimension in the other instruments [6]. Although the concepts of social isolation, loneliness, social network, social support, and social participation were identified through this effort, the review did not propose an overarching theoretical framework of social frailty.

Within Asia, where many societies are aging rapidly, social frailty is especially germane due to challenges such as changing structures and attitudes towards older members of the familial unit [7], social participation, and environments. Prior Asian studies examining the impact of social frailty have done so using brief questionnaires of 5–7 items which are primarily modeled after the 5-item Fried’s frailty phenotype score. For instance, using a 5-item social frailty questionnaire, studies in Japan and Korea reported that social frailty increased the risk of disability and depressed mood, and was associated with cognitive and physical deficits in older adults [8,9,10]. Similarly, social frailty measured using a 5-item scale was associated with subjective memory decline, cognitive impairment, depression, and physical functioning, and predicted mortality in China [11]. A Singapore study utilized a 7-item index to demonstrate that social frailty increased the prevalence and incidence of functional disability, independently and when combined with physical frailty [12].

Though these studies revealed significant associations with the measured outcomes, they were not premised on a conceptual framework of social frailty. The construct validity of the social frailty brief scales was also not delineated through empirical statistical techniques such as factor analysis. Furthermore, these studies mainly comprised less robust community-dwelling older adults, such that the relationship between social and physical aspects of frailty may potentially be confounded. In addition, other salient outcomes such as nutrition, physical performance, physical activity, and life–space mobility were not studied. Lastly, in Asian societies where traditional family values are cherished, commensality (the act of eating together) is generally considered a form of social engagement during mealtimes, with family or friends. However, earlier Asian studies did not include any item that pertained to ‘eating alone’ when examining the association of social frailty with adverse outcomes [13].

We therefore conducted this study to examine the independent association of social frailty with a comprehensive range of intermediary outcomes in a representative cohort of non-frail community-dwelling Asian older adults. There are two parts to our study. Firstly, using validated items identified from prior Asian studies, we performed exploratory factor analysis (EFA) to derive a social frailty scale grounded in Bunt’s proposed conceptual framework. Next, using the empirically developed social frailty scale, we studied the association of social pre-frailty and social frailty, independently of physical frailty, with outcomes of mood, nutrition, physical performance, physical activity and life–space mobility. Through this, we aim to anchor understanding of the impact of social frailty on pertinent outcomes in an Asian setting from a theory-based framework.

## 2. Materials and Methods

### 2.1. Study Population

The “Longitudinal Assessment of Biomarkers for characterization of early Sarcopenia and Osteosarcopenic Obesity in predicting frailty and functional decline in community-dwelling Asian older adults Study” (GeriLABS 2) is a prospective cohort study involving cognitively intact and functionally independent adults aged 50 years and older residing within the community. We recruited 230 participants from December 2017 to March 2019. In this cross-sectional analysis, one participant was excluded due to missing values in the data. The final sample comprised 229 participants who completed all baseline clinical assessments. Participants were included if they were (i) aged 50 to 99 years at study enrolment, (ii) community-dwelling, (iii) independent in both activities of daily living (ADLs) and instrumental ADLs, and (iv) non-frail as defined by the FRAIL criteria [14]. We excluded participants with a known history of dementia or evidence of cognitive impairment (modified Chinese version of Mini-Mental State Examination (CMMSE) score ≤21) [15]; who are unable to walk 8-m independently; and living in a sheltered or nursing home. All participants provided written informed consent for inclusion before they participated in the study. The study was conducted in accordance with the Declaration of Helsinki, and the protocol was approved by the Domain Specific Review Board of the National Healthcare Group (DSRB Ref: 2017/00850).

### 2.2. Clinical Assessment

We collected demographic data and comorbid vascular risk factors. Anthropometric measurements including standing height and body weight were measured to calculate body mass index, in addition to waist, mid-arm, and calf circumferences. Cognitive performance was assessed using the modified Chinese version of Mini-Mental State Examination (CMMSE). Functional status was evaluated using Barthel’s basic activities of daily living (BADL) index [16] and Lawton and Brody’s instrumental ADL (IADL) index [17]. Physical frailty was assessed using the modified Fried phenotypic criteria [18]. The modified Fried criteria were operationalized as follows [19]: (1) Body mass index less than 18.5; (2) handgrip strength <26 kg for men and <18 kg for women measured using a hydraulic hand dynamometer (North Coast Exacta™ Hydraulic Hand Dynamometer; North Coast Medical, Inc., Morgan Hill, CA, USA) [20]; (3) usual gait speed <0.8 m/s on the 3-m walk test; (4) low physical activity defined using the pentile cutoff of ≤29 on the Frenchay Activities Index [21]; and (5) fatigue endorsed on either of two questions from the Center for Epidemiologic Studies–Depression Scale (CES-D) modified to assess fatigue. The five items were added to yield a total score (range 0–5), which corresponded respectively to a status of robust (0), pre-frail (1–2), and frail (3–5).

### 2.3. Social Frailty Questionnaire Items

We performed a literature search on social frailty in Asia for studies with social frailty scales published before November 2017, supplemented by a reference search of retrieved articles and recommendations from experts in the field. The items identified from these published Asian studies were used in our analysis. Altogether, nine items were identified, comprising five items from Makizako et al. [8] and Tsutsumimoto et al. [9]; two items from Tanaka et al. [13]; and two items from Teo et al. [12]. The combined 9-item social frailty questionnaire was administered, with equal weightage of one point assigned to each item: (1) “Do you live alone?”; (2) “Do you go out less frequently compared with last year?”; (3) “Do you sometimes visit your friends?”; (4) “Do you feel you are helpful to friends or family?”; (5) “Do you talk with someone every day?”; (6) “Do you turn to family or friends for advice?”; (7) “Do you eat with someone at least one time in a day?”; (8) “Do you have someone to confide in?”; and (9) “Are you limited by your financial resources to pay for needed medical service?”. From Teo et al., questions demonstrating duplication such as infrequent contact and social activities were removed. Similarly, demographic questions on education and housing type were removed and captured under clinical assessment.

### 2.4. Outcome Measures

Mood was assessed using the 15-item Geriatric Depression Scale (GDS), with a locally validated cutoff score of ≥4 to distinguish presence of depressive symptoms [22]. Nutrition was measured with the Mini Nutritional Assessment (MNA), with a cutoff score of <24 indicating malnutrition risk [23]. Other nutritional parameters assessed included the Simplified Nutritional Appetite Questionnaire (SNAQ) [24], as well as Vitamin D and albumin levels. Physical performance was measured using the Short Physical Performance Battery (SPPB), which comprised balance, gait speed, and chair stand tests; a cutoff of <10 denoted poor physical performance [25]. Physical activity was derived from the International Physical Activity Questionnaire (IPAQ) [26] after converting responses to Metabolic Equivalent Task (MET) minutes per week. Life–space mobility was measured using the Life–Space Assessment (LSA) [27] comprising spatial areas, frequency, and level of independence required. There are five life–space levels, which represented activities outside the bedroom, home, neighborhood, town, and beyond respectively.

### 2.5. Statistical Analyses

To ascertain the factor structure of the combined 9-item Social Frailty questionnaire, we conducted exploratory factor analysis (EFA) using the Kaiser–Meyer–Olkin (KMO) statistic as a measure of sampling adequacy and the Bartlett test of sphericity to ascertain necessity to perform a factor analysis. We performed principal component analysis with varimax rotation to ascertain the underlying factor structure. The number of factors to be retained was determined by parallel analysis, a more robust and accurate method of factor retention that was less likely to overestimate the number of factors [28]. We eliminated items with loadings <0.4. The retained factors were interpreted using Bunt’s conceptual framework.

Using the resultant factors and items in the Social Frailty questionnaire, we derive optimal cutoffs to categorize participants into three subgroups: social non-frailty, social pre-frailty, and social frailty. The cutoffs were empirically determined based on distribution to match the trend seen in earlier Asian studies. We performed univariate analyses to compare baseline demographics, cognitive performance, functional and frailty status, and outcome measures of mood, nutrition, physical performance, physical activity, and life–space mobility across the three subgroups. We used a one-way analysis of variance with Bonferroni correction for post-hoc comparison and Kruskal–Wallis test respectively for parametric and non-parametric continuous variables, and a Chi-square test for categorical variables.

**Figure 2:**
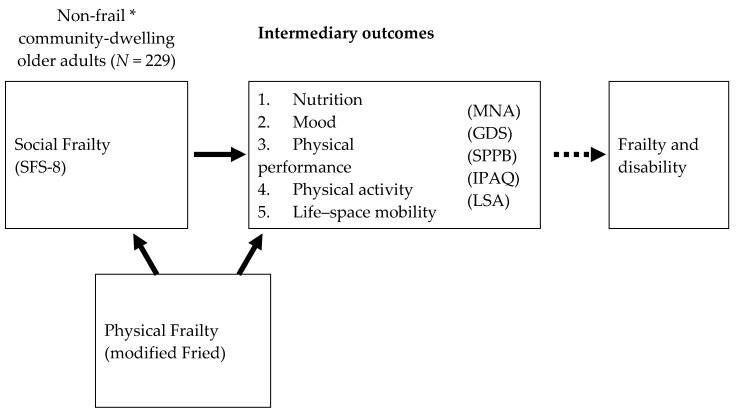
Conceptual diagram of study. This figure illustrates the relationship between social frailty (independent variable) with intermediary outcome variables of nutrition, mood, physical performance, physical activity and life space mobility, which in turn can lead to downstream frailty and disability (not measured in this study). Physical frailty as measured by modified Fried criteria is a confounder variable which can affect both social frailty and intermediary outcomes. * defined by FRAIL criteria; GDS, Geriatric Depression Scale; IPAQ, International Physical Activity Questionnaire; LSA, Life–Space Assessment; MNA, Mini Nutritional Assessment; SFS-8, 8-item Social Frailty Scale; SPPB, Short Physical Performance Battery.

To determine the independent association of social pre-frailty and social frailty to our pre-specified outcomes, we performed hierarchical logistic regression, adjusting for age, gender, variables which were significant on univariate analysis, and physical frailty (modified Fried phenotypic criteria). Due to low numbers in the frail category by Fried scoring, we used the total score instead in the logistic regression model. Cutoffs for GDS, MNA, and SPPB were defined using validated cutoffs as described, while low physical activity and low life–space mobility were defined using the cohort quintile cutoffs of IPAQ < 2826 METs and LSA < 76 respectively. In Model 1, we adjusted for age, gender, and other significant variables. In Model 2, we adjusted for physical frailty in addition to the variables in Model 1. For comparison, we performed logistic regression with physical frailty as the independent variable adjusting for age, gender and significant variables (Figure 2).

Statistical analyses were performed using IBM SPSS Statistics version 23.0 (IBM Corporation, Armonk, NY, USA). All statistical tests were two-tailed, with *p* < 0.05 considered statistically significant.

## 3. Results

### 3.1. Demographic and Clinical Characteristics

We studied 229 participants with a mean age of 67.22 ± 7.43 years, of which 167 (72.6%) were females (Table 1). Participants received a mean 10.73 ± 4.36 years of education, with the majority (71.2%) residing in public housing apartments. Comorbidities include hypertension (35.8%), hyperlipidemia (56.8%), and Type II Diabetes Mellitus (14.4%). The high cognitive score (CMMSE, mean ± SD: 26.12 ± 1.73) and functional status (BADL and IADL had respective median scores of 100 and 23, corresponding to the maximum score) attested to the relatively robust health of the participants. There were 196 (85.6%) robust and 33 (14.4%) pre-frail participants identified using the FRAIL criteria. Based on the modified Fried criteria, only two (0.9%) participants were physically frail, with 95 (41.5%) physically pre-frail and 132 (57.6%) robust.

### 3.2. Factor Analysis and Questionnaire Items

Factor analysis was appropriate as the KMO statistic was 0.586, and the Bartlett test of sphericity was 186 (*p* < 0.0001). We chose a three-factor solution, as per the optimal number recommended by parallel analysis, which accounted for 50.5% of total variance (Table 2). Taking reference from Bunt’s conceptual framework, the first factor (22.4% of variance) had three items which represented social resources; the second factor (15.5% of variance) had three items corresponding to social activities and financial resource; and the third factor (12.6% of variance) with two items denoted social need fulfilment. The question “Do you feel you are helpful to friends or family?” was eliminated due to its low loading of 0.490 and non-discriminatory nature of having only six (2.6%) participants endorsing this item. Thus, there were eight items in the final version of the Social Frailty Scale (SFS-8). The items were summed to yield a total score which was used to categorize participants into three subgroups of social non-frailty (SNF; 0–1 point), social pre-frailty (SPF; 2–3 points) and social frailty (SF; 4–8 points). Using these cutoffs to categorize the subgroups, 146 (63.8%) of participants were classified as SNF, 66 (28.8%) as SPF, and 17 (7.4%) as SF, which is consistent with the distribution trend seen in previous Asian studies [8,9].

### 3.3. Comparison across Social Frailty Subgroups

Comparing baseline characteristics across the three subgroups (Table 1), age increased and was significantly higher in SF subgroup compared with SNF. Educational level decreased and was significantly lower in SPF compared with SNF. There was no significant difference in gender, housing type, and anthropometric measurements. Among comorbidities, only hypertension was significantly different across the three subgroups. BADL was significant lower in the SF subgroup but there was no significant difference in CMMSE and IADL. Modified Fried score was also significantly higher in SF and SPF subgroups compared with SNF, corresponding to the higher proportion of physical pre-frailty and frailty observed in these two subgroups.

For the 8-item Social Frailty Scale (SFS-8), total score increased significantly across the three subgroups (*p* < 0.001), with the post-hoc analysis indicating significantly higher scores in SF subgroup compared with SPF and SNF, and SPF compared with SNF (Table 3). Likewise, all factor scores were significantly different across the three subgroups (all *p* < 0.001), with significant post-hoc differences when comparing SF with both SPF and SNF, and SPF compared with SNF.

### 3.4. Associations between Social Frailty and Outcome Measures

Across the three subgroups (Table 4), GDS score was significantly higher comparing SF with both SPF and SNF, and SPF with SNF (SNF 0 (interquartile range, IQR: 0–1.00) vs. SPF 1.00 (IQR: 0–2.00) vs. SF 2.00 (IQR: 1.00–3.00), *p* < 0.001). For nutrition, MNA, SNAQ, and albumin level were significant (all *p* < 0.05), with post-hoc analyses indicating significant differences between SPF and SNF for MNA and albumin level. For physical performance, SPPB, gait speed, 5-time repeated chair stand, and handgrip strength were all significant across the three subgroups (all *p* < 0.05). Post-hoc comparisons showed the SF and SPF groups performing significantly worse than SNF in SPPB and 5-time repeated chair stand. Gait speed was significantly slower in SF compared to SNF, while handgrip strength was significantly lower in SPF when compared to SNF.

In terms of physical activity and life–space, IPAQ was significantly different across the three subgroups (*p* = 0.001), with post-hoc comparisons revealing significantly lower activity in SF and SPF compared to SNF. Life–space mobility was significantly lower for life–space Levels 2 and 5, corresponding to being in areas outside one’s home and places outside one’s town (*p* = 0.026 and *p* = 0.006, respectively), with significant difference between SPF and SNF in post-hoc analyses.

### 3.5. Logistic Regression Analysis for Outcomes

We performed logistic regression analyses to examine the independent association of social frailty with outcome measures (Table 5). In Model 1, adjusting for age, gender, education, hypertension and albumin, SPF was significantly associated with poor physical performance measured by SPPB (odds ratio, OR = 7.66, 95% confidence interval, CI = 1.43–41.14) and low physical activity (OR = 3.66, 95% CI = 1.67–8.02), whereas SF was significantly associated with low mood (OR = 6.88, 95% CI = 1.23–38.66); malnutrition risk (OR = 11.13, 95% CI = 1.91–64.97); poor physical performance (OR = 17.51, 95% CI = 2.63–116.58); and low physical activity (OR = 4.46, 95% CI = 1.37–14.54). There was no significant association with life–space. When additionally adjusted for physical frailty in Model 2, the significant association with poor physical performance and low physical activity remained for SPF (OR range: 3.10 to 6.22), and with low mood, malnutrition risk, poor physical performance, and low physical activity for SF (OR range: 3.58 to 13.97).

We repeated logistic regression analyses to examine the association of physical frailty with outcome measures. Physical frailty was significantly associated with malnutrition risk (OR = 3.47, 95% CI = 1.33–9.05), low physical activity (OR = 1.78, 95% CI = 1.04–3.07), and decreased life–space (OR = 2.19, 95% CI = 1.26–3.81), but not with low mood or poor physical performance.

## 4. Discussion

In the present study, using a theory-guided social frailty scale that is grounded in the Bunt conceptual framework, we build upon growing body of evidence about the paramount importance of social frailty by demonstrating the independent associations of SPF and SF with mood, nutrition, physical performance, and physical activity in non-frail community-dwelling older adults. Even after adjusting for physical frailty, both SPF and SF were associated with poor physical performance and low physical activity, with SF also associated with low mood and malnutrition. This increase in odds from SNF to SF attests to a dose–response relationship for these outcomes, lending credence to the validity of our findings. With the significant prevalence of SPF and SF at 28.8% and 7.4%, respectively, in our cohort of non-frail older adults, the independent associations of social frailty with intermediary outcomes which precede the onset of frailty and disability corroborate the contributory role of social components towards increased vulnerability in older adults [6] and emphasize the importance of evaluating social dimensions as part of a comprehensive geriatric assessment.

The theoretical framework and definition of social frailty proposed by Bunt et al. [5] reinforced our approach in understanding this complex construct. From the initial 9-item questionnaire, we excluded the item “Do you feel you are helpful to friends or family?” despite it being an element under Bunt’s ‘general resources’ category. The non-discriminatory response with this item may either represent under-reporting due to desirability bias or the lack of relevance of feeling helpful to friends or family in the overall construct of social frailty. The resultant SFS-8 items cohered to our three factors of ‘social resources’, ‘social activities and financial resource’, and ‘social need fulfilment’, which addressed the various components when mapped onto Bunt’s social frailty concept. Interestingly, the items grouped under Factor 2 (Table 2) may suggest a relationship between constraints on financial resources for medical services with social activities of going out and eating with someone. Alluding further to Bunt’s ‘general resources’ category, the trends observed in baseline characteristics, such as education, housing, BADL performance and cognitive performance moving from SNF to SF subgroups, also support the known-group validity of the SFS-8 cutoffs used to define subgroups. While a recent study considered the Bunt’s social frailty conceptual model when using a 4-item social frailty assessment tool to evaluate the impact on incident disability and mortality [29], it only had one item per Bunt category and did not comprehensively delineate the components that underpin social frailty [5]. Notwithstanding differences in countries and cultures, our study provides a starting point for a theory-driven approach with reference to Asian evidence, in examining the impact of social frailty on salient outcomes in older adults.

As far as we are aware, this is the first study to examine the association between social frailty and nutrition in older adults, illuminating the magnitude of its impact on malnutrition risk. Previous studies examining the relationship between frailty and nutrition [30,31] placed heavy emphasis only on the physical aspect of frailty. In addition, our results showing the association between social frailty and depressive symptoms paralleled similar findings from a previous study [11]. Indeed, insights from these findings can explicate the potential protective role of commensality against social frailty. Besides leading to depressive mood and enhancing feeling of loneliness [32], eating alone among older adults can also result in lower food diversity [30] and poorer nutritional status [33], due to the lack of social companionship during mealtimes [34]. In the communal dining culture of Asia, commensality thus serves as an important avenue for socialization where older adults enjoy interactions and gain valuable opportunities for companionship [35,36]. In our study, although the majority (88.6%) of participants lived with others, it is disconcerting that 24.5% constantly ate alone and 28.4% did not talk to others daily. The reasons for these findings are unclear and warrant further studies to ascertain if the prevalence of eating alone and decreased social interaction is even higher amongst less robust populations of community-dwelling older adults.

As an antecedent to functional decline and/or disability, physical activity is considered an important interventional target in the prevention of frailty in older adults [37,38]. Building upon emerging evidence that social frailty is an important risk factor of physical deficits [9] and disability [12] that may lead to the subsequent development of physical frailty in non-frail older adults [39], our results showing significant associations of both SPF and SF with physical performance and physical activity mirrored this trend. Although physical performance and physical activity have been reported to be independently associated with life–space mobility in older adults [40,41], we did not find a similar association of social frailty with life–space mobility. Interestingly, in our non-frail cohort (defined by FRAIL score), social frailty was associated with adverse outcomes even after adjustment for physical frailty (modified Fried), and conferred increased odds for GDS, MNA, SPPB, and IPAQ. 

Taken together, our results suggest the preeminence of social frailty in assessing the risk profile of adverse health outcomes in robust populations of older adults. Notably, three of the outcomes (GDS, MNA, and SPPB) associated with social frailty are components of the novel construct of intrinsic capacity, which emphasizes on the more positive attributes of reserves and residual capacities, as opposed to deficits and limitations accumulated with aging in frailty [42,43]. Thus, consistent with a life-course approach towards healthy aging, our results suggest the possible role of upstream community-based interventions to target the deleterious impact of social frailty in non-frail older adults, such as programs that promote social interaction, engagement in physical activities, and sharing of nutritious meals to build social capital and intrinsic reserves through social networking and community participation [44,45]. Future studies should further delineate the longitudinal relationship between social frailty and intrinsic capacity and whether social frailty may be the forme fruste of an underlying age-related or even pathological process.

In addition, although social determinants of health are often the main driver of health inequalities within and between countries [46], better recognition of these conditions together with frailty has been mooted [47]. Indeed, studies have shown that not only objective measures of socioeconomic status such as education, employment, and income impacted on frailty trajectories [48], lower subjective social status was also associated with a higher incidence of frailty in men [49]. The specific relationship with social frailty in these studies, however, remains unclear. The increased odds of adverse outcomes associated with social frailty as illuminated in our findings, coupled with many socioeconomic elements contributing towards social frailty as outlined in Bunt’s framework [5], become especially pertinent during the current COVID-19 climate. The pandemic has galvanized the world into unprecedented efforts of instituting physical distancing, such that social frailty can be amplified due to the secondary effects of social isolation in many older adults [50]. Other examples of how pandemic control measures may exacerbate inequalities include the withdrawal of ‘non-essential’ services that provide support for older adults living alone or with cognitive impairment, or the inequitable access to digital tools to mitigate social isolation amongst older adults from lower socioeconomic background who may have lower digital literacy. Further studies are therefore warranted to better understand and address the social gradient and inequalities that may aggravate the impact of social frailty. 

This study has several limitations. Firstly, the cross-sectional analysis precludes definitive conclusions about causality as reverse causation cannot be excluded. A causal relationship should be elucidated in well-conducted prospective studies to examine the longitudinal impact of social frailty on salient outcomes. Secondly, our study comprised predominantly Chinese older adult participants who were robust and higher functioning. Thus, our findings may not be generalizable to non-Chinese Asian settings with more frail older adults. Thirdly, we recognize that Bunt’s social frailty model supported a further understanding into self-management abilities by which one gains or maintains resources which are necessary for social need fulfilment and its higher-level outcome of subjective wellbeing, both of which were not examined in this study. Finally, we utilized questionnaire items from previous Asian studies to operationalize social frailty. Nonetheless, it remains a complex construct which will require qualitative and mixed method approaches to explore in greater depth. 

## 5. Conclusions

In conclusion, our study supports the incorporation of a theory-grounded conceptual model of social frailty. The factors in our Social Frailty Scale cohere with social resources, general resources, social behavior/activities, and fulfilment of basic social needs. We demonstrated the association of social frailty, independently of physical frailty, with important outcomes of mood, nutrition, physical performance and physical activity in healthy community-dwelling older adults. Our findings highlight the need for further studies to consider the social dimension of frailty, design upstream interventions to target the impact of social frailty and address social gradient and inequalities. 

## Figures and Tables

**Table 1:** Baseline characteristics of study cohort (*n* = 229).

Variables	Total	Social Non-Frailty	Social Pre-Frailty	Social Frailty	*p*-Value
	*n* = 229	*n* = 146 (63.8%)	*n* = 66 (28.8%)	*n* = 17 (7.4%)	
Demographics					
Age, years	67.22 ± 7.43	66.03 ± 6.66	68.61 ± 7.83	72.12 ± 9.49 ^a^	0.001
Female, *n* (%)	167 (72.6)	102 (69.9)	52 (78.8)	12 (70.6)	0.397
Chinese ethnicity, *n* (%)	212 (92.6)	134 (91.8)	62 (93.9)	16 (94.1)	0.941
Education, years	10.73 ± 4.36	11.49 ± 4.43	9.48 ± 3.91 ^b^	9.12 ± 4.11	0.002
Housing, *n* (%)					0.083
1–2 room	7 (3.1)	2 (1.4)	4 (6.1)	1 (5.9)	
3–5 room	156 (68.1)	95 (65.1)	47 (71.2)	14 (82.4)	
Executive & above	66 (28.8)	49 (33.6)	15 (22.7)	2 (11.8)	
Co-morbidities					
Hypertension, *n* (%)	82 (35.8)	43 (29.5)	31 (47.0)	8 (47.1)	0.029
Hyperlipidemia, *n* (%)	130 (56.8)	85 (58.2)	35 (53.0)	10 (58.8)	0.767
Diabetes, *n* (%)	33 (14.4)	23 (15.8)	10 (15.2)	0 (0)	0.212
Ischemic Heart Disease, *n* (%)	5 (2.2)	4 (2.7)	1 (1.5)	0 (0)	0.695
Atrial Fibrillation, *n* (%)	7 (3.1)	5 (3.4)	2 (3.1)	0 (0)	0.740
Stroke, *n* (%)	4 (1.7)	2 (1.4)	1 (1.5)	1 (5.9)	0.399
Peripheral Vascular Disease, *n* (%)	0 (0)	0 (0)	0 (0)	0 (0)	
Smoking, *n* (%)	19 (8.3)	12 (8.2)	6 (9.1)	1 (5.9)	0.911
Vascular Risk Factor score	1.22 ± 1.10	1.19 ± 1.14	1.30 ± 1.07	1.18 ± 0.95	0.783
Osteoporosis, *n* (%)	63 (27.5)	41 (28.1)	17 (25.8)	5 (29.4)	0.925
Anthropometry					
Weight, kg	59.45 ± 9.69	59.86 ± 9.18	59.26 ± 11.05	56.70 ± 8.25	0.438
BMI, kg/m^2^	23.89 ± 3.23	23.93 ± 2.99	23.97 ± 3.79	23.20 ± 2.99	0.661
Waist circumference, cm	85.34 ± 9.32	85.17 ± 8.46	85.63 ± 11.49	85.66 ± 7.16	0.935
Mid arm circumference, cm	27.65 ± 3.00	27.67 ± 2.65	27.90 ± 3.34	26.44 ± 4.26	0.200
Calf circumference, cm	34.78 ± 3.20	35.02 ± 2.90	34.46 ± 3.85	33.94 ± 2.78	0.267
Cognitive performance					
CMMSE, max 28	26.12 ± 1.73	26.25 ± 1.67	26.05 ± 1.85	25.35 ± 1.66	0.120
Functional status					
BADL ^, max 100	100.00 (95.00–100.00)	100.00 (100.00–100.00)	100.00 (95.00–100.00)	95.00 (92.50–100.00) ^a^	0.004
IADL ^, max 23	23.00 (22.00–23.00)	23.00 (22.75–23.00)	23.00 (22.00–23.00)	23.00 (22.00–23.00)	0.152
Frailty status					
FRAIL score, max 5	0.16 ± 0.41	0.13 ± 0.34	0.21 ± 0.51	0.24 ± 0.56	0.307
Robust, *n* (%)	196 (85.6)	127 (87.0)	55 (83.3)	14 (82.4)	
Pre-frail, *n* (%)	33 (14.4)	19 (13.0)	11 (16.7)	3 (17.6)	
Frail, *n* (%)	0 (0)	0 (0)	0 (0)	0 (0)	
Modified Fried ^, max 5	0 (0–1.00)	0 (0–1.00)	0 (1.00–1.00) ^b^	1.00 (0–1.00) ^a^	0.000
Robust, *n* (%)	132 (57.6)	99 (67.8)	28 (42.4)	5 (29.4)	
Pre-frail, *n* (%)	95 (41.5)	47 (32.2)	37 (56.1)	11 (64.7)	
Frail, *n* (%)	2 (0.9)	0 (0)	1 (1.5)	1 (5.9)	

Mean ± SD unless otherwise indicated; ^ Median (interquartile range, IQR); BADL, basic activities of daily living; BMI, body mass index; CFAB, Chinese Frontal Assessment Battery; CMMSE, Chinese Mini-Mental State Examination; IADL, Instrumental activities of daily living. ^a^ Post-hoc, *p* < 0.05 compared with social non-frailty; ^b^ Post-hoc, *p* < 0.05 compared with social non-frailty.

**Table 2:** Exploratory factor analysis (EFA; varimax rotation, three-factor extraction, loadings >0.400).

Social Frailty Combined Questionnaire Items	Factors			
	Mean ± SD	1	2	3
Factor 1: Social resources				
Do you sometimes visit your friends?	0.14 ± 0.35	0.616		
Do you turn to family or friends for advice?	0.16 ± 0.36	0.816		
Do you have someone to confide in?	0.15 ± 0.36	0.720		
Factor 2: Social activities and financial resource				
Do you go out less frequently compared with last year?	0.15 ± 0.36		0.618	
Do you eat with someone at least one time in a day?	0.24 ± 0.43		0.580	
Are you limited by your financial resources to pay for needed medical service?	0.08 ± 0.27		0.635	
Do you feel you are helpful to friends or family? *	0.03 ± 0.16		0.490	
Factor 3: Social need fulfilment				
Do you live alone?	0.11 ± 0.32			0.750
Do you talk with someone every day?	0.28 ± 0.45			0.634
Eigenvalue		2.02	1.40	1.13
Percentage of explained variance		22.4	15.5	12.6

* Item was removed from the Social Frailty Scale.

**Table 3:** Factors and items by social frailty status.

Factors and Items	Total	Social Non-Frailty	Social Pre-Frailty	Social Frailty	*p*-Value
	*n* = 229	*n* = 146	*n* = 66	*n* = 17	
Social Frailty factors, mean ± SD					
Total score (max 8)	1.31 ± 1.40	0.44 ± 0.50	2.41 ± 0.50 ^c^	4.59 ± 1.12 ^a,b^	0.000
Factor 1: Social resources, max 3	0.45 ± 0.80	0.10 ± 0.31	0.85 ± 0.90 ^c^	1.82 ± 1.13 ^a,b^	0.000
Factor 2: Social activities and financial resource, max 3	0.47 ± 0.70	0.20 ± 0.40	0.80 ± 0.73 ^c^	1.53 ± 1.07 ^a,b^	0.000
Factor 3: Social need fulfilment, max 2	0.40 ± 0.61	0.14 ± 0.35	0.76 ± 0.66 ^c^	1.24 ± 0.75 ^a,b^	0.000
Individual items, *n* (%)					
Visiting friends sometimes (no)	32 (14.0)	6 (4.1)	17 (25.8)	9 (52.9)	0.000
Turning to family or friends for advice (no)	36 (15.7)	4 (2.7)	20 (30.3)	12 (70.6)	0.000
Having someone to confide in (no)	34 (14.8)	5 (3.4)	19 (28.8)	10 (58.8)	0.000
Going out less frequently compared with the last year (yes)	34 (14.8)	9 (6.2)	15 (22.7)	10 (58.8)	0.000
Eating with someone at least one time in a day (no)	56 (24.5)	18 (12.3)	28 (42.4)	10 (58.8)	0.000
Limited by financial resources to pay for needed medical service (yes)	18 (7.9)	2 (1.4)	19 (15.2)	6 (35.3)	0.000
Living alone (yes)	26 (11.4)	5 (3.4)	14 (21.2)	7 (41.2)	0.000
Talking to someone every day (no)	65 (28.4)	15 (10.3)	36 (54.5)	14 (82.4)	0.000

^a^ Post-hoc, *p* < 0.05 compared with social non-frailty; ^b^ Post-hoc, *p* < 0.05 compared with social pre-frailty; ^c^ Post-hoc, *p* < 0.05 compared with social non-frailty.

**Table 4:** Association of social frailty with outcomes.

Outcome Variables	Total	Social Non-Frailty	Social Pre-Frailty	Social Frailty	*p*-Value
	*n* = 229	*n* = 146 (63.8%)	*n* = 66 (28.8%)	*n* = 17 (7.4%)	
Mood					
GDS ^, max 15	1.00 (0–2.00)	0 (0–1.00)	1.00 (0–2.00) ^c^	2.00 (1.00–3.00) ^a,b^	0.000
Nutrition					
MNA total, max 30	27.20 ± 1.86	27.49 ± 1.65	26.74 ± 2.05 ^c^	26.44 ± 2.37	0.005
SNAQ total, max 20	15.79 ± 1.51	15.96 ± 1.35	15.56 ± 1.78	15.18 ± 1.43	0.045
Vitamin D, ng/mL	30.58 ± 8.79	30.40 ± 8.87	30.74 ± 7.59	31.59 ± 12.31	0.857
Albumin, g/l	41.16 ± 2.57	41.47 ± 2.47	40.47 ± 2.66 ^c^	41.18 ± 2.67	0.032
Physical performance					
SPPB ^, max 12	12.00 (11.00–12.00)	12.00 (12.00–12.00)	12.00 (11.00–12.00) ^c^	11.00 (9.50–12.00) ^a^	0.000
Gait speed, m/s	1.17 ± 0.23	1.20 ± 0.23	1.14 ± 0.22	1.05 ± 0.18 ^a^	0.014
5-time repeated chair stand, sec	9.46 ± 3.03	8.73 ± 2.74	10.45 ± 3.07 ^c^	11.92 ± 3.01 ^a^	0.000
Handgrip strength, kg	23.51 ± 7.14	24.75 ± 7.13	21.47 ± 6.68 ^c^	20.76 ± 6.80	0.002
Physical activity and life–space					
IPAQ, METs	5042.45 ± 2402.70	5473.98 ± 2364.50	4321.39 ± 2278.25 ^c^	4135.75 ± 2407.14 ^a^	0.001
Life–space Level 1, max 8	7.97 ± 0.30	7.99 ± 0.17	7.94 ± 0.49	8.00 ± 0.00	0.526
Life–space Level 2, max 16	15.35 ± 2.17	15.64 ± 1.62	14.85 ± 2.87 ^c^	14.82 ± 2.74	0.026
Life–space Level 3, max 24	18.71 ± 6.90	19.19 ± 6.85	17.39 ± 6.90	19.76 ± 6.96	0.171
Life–space Level 4, max 32	21.46 ± 8.51	21.19 ± 8.00	21.52 ± 9.03	23.53 ± 10.76	0.564
Life–space Level 5, max 40	26.79 ± 9.68	28.30 ± 9.55	24.28 ± 9.28 ^c^	23.53 ± 9.96	0.006
Life–space Total, max 120	90.34 ± 17.64	92.26 ± 16.31	86.27 ± 18.71	89.65 ± 22.32	0.071

Mean ± SD unless otherwise indicated; ^ Median (IQR); GDS, Geriatric Depression Scale; IPAQ, International Physical Activity Questionnaire; MET; Metabolic Equivalent Task; MNA, Mini Nutritional Assessment; SNAQ, Short Nutritional Assessment Questionnaire; SPPB, Short Physical Performance Battery. ^a^ Post-hoc, *p* < 0.05 compared with social non-frailty; ^b^ Post-hoc, *p* < 0.05 compared with social pre-frailty; ^c^ Post-hoc, *p* < 0.05 compared with social non-frailty.

**Table 5:** Logistic regression models for social and physical frailty.

Outcome Variables	Unadjusted		Model 1		Model 2	
	Odds Ratio (95% CI)	*p*-Value	Odds Ratio (95% CI)	*p*-Value	Odds Ratio (95% CI)	*p*-Value
GDS						
1. Social Non-Frailty	Reference		Reference		Reference	
Social Pre-Frailty	1.28 (0.36–4.54)	0.701	1.24 (0.30–5.08)	0.765	1.14 (0.26–4.89)	0.864
Social Frailty	4.26 (0.99–18.32)	0.052	6.88 (1.23–38.66)	0.028	6.32 (1.12–35.80)	0.037
2. Modified Fried	1.16 (0.53–2.55)	0.704	1.32 (0.53–3.26)	0.552	-	
MNA						
1. Social Non-Frailty	Reference		Reference		Reference	
Social Pre-Frailty	3.55 (0.97–13.03)	0.056	3.57 (0.87–14.69)	0.078	2.24 (0.48–10.46)	0.307
Social Frailty	7.61 (1.54–37.47)	0.013	11.13 (1.91–64.97)	0.007	8.35 (1.18–59.22)	0.034
2. Modified Fried	2.94 (1.45–5.99)	0.003	3.47 (1.33–9.05)	0.011	-	
SPPB						
1. Social Non-Frailty	Reference		Reference		Reference	
Social Pre-Frailty	8.54 (1.72–42.33)	0.009	7.66 (1.43–41.14)	0.018	6.22 (1.09–35.43)	0.039
Social Frailty	22.15 (3.70–132.66)	0.001	17.51 (2.63–116.58)	0.003	13.97 (1.97–98.90)	0.008
2. Modified Fried	2.94 (1.45–5.99)	0.003	1.75 (0.76–4.06)	0.192	-	
IPAQ						
1. Social Non-Frailty	Reference		Reference		Reference	
Social Pre-Frailty	2.91 (1.43–5.93)	0.003	3.66 (1.67–8.02)	0.001	3.10 (1.37–7.04)	0.007
Social Frailty	3.65 (1.20–11.01)	0.022	4.46 (1.37–14.54)	0.013	3.58 (1.06–12.04)	0.040
2. Modified Fried	1.81 (1.14–2.88)	0.012	1.78 (1.04–3.07)	0.037	-	
LSA Total						
1. Social Non-Frailty	Reference		Reference		Reference	
Social Pre-Frailty	2.23 (1.10–4.55)	0.027	2.02 (0.94–4.31)	0.070	1.49 (0.66–3.37)	0.337
Social Frailty	1.83 (0.55–6.16)	0.328	1.72 (0.48–6.10)	0.403	1.16 (0.30–4.56)	0.829
2. Modified Fried	2.20 (1.37–3.53)	0.001	2.19 (1.26–3.81)	0.005	-	

GDS, Geriatric Depression Scale; IPAQ, International Physical Activity Questionnaire; LSA, Life–Space Assessment; MNA, Mini Nutritional Assessment; SPPB, Short Physical Performance Battery. Model 1: Adjusted for age, gender, education, hypertension, and albumin. Model 2: Adjusted for Model 1 plus physical frailty.
